# Monoclonal antibody-mediated neutralization of SARS-CoV-2 in an IRF9-deficient child

**DOI:** 10.1073/pnas.2114390118

**Published:** 2021-10-26

**Authors:** Romain Lévy, Peng Zhang, Paul Bastard, Karim Dorgham, Isabelle Melki, Alice Hadchouel, George C. Hartoularos, Bénédicte Neven, Martin Castelle, Charlotte Roy, Tom Toin, Laureline Berteloot, Lucy Bizien, Hanène Abid, Marianne Burgard, Nadhira Houhou-Fidouh, Flore Rozenberg, Emmanuelle Jouanguy, Chun Jimmie Ye, Guy Gorochov, Qian Zhang, Jean-Laurent Casanova

**Affiliations:** ^a^Laboratory of Human Genetics of Infectious Diseases, Necker Branch, INSERM, Necker Hospital for Sick Children 75015 Paris, France;; ^b^Imagine Institute, University of Paris, Paris 75015, France;; ^c^Pediatric Hematology-Immunology and Rheumatology Unit, Necker Hospital for Sick Children, Assistance Publique–Hôpitaux de Paris, Paris 75015, France;; ^d^St. Giles Laboratory of Human Genetics of Infectious Diseases, Rockefeller Branch, The Rockefeller University, New York, NY 10065;; ^e^Sorbonne Université, INSERM, Centre for Immunology and Microbial Infections-Paris, Pitié-Salpêtrière Hospital, Assistance Publique–Hôpitaux de Paris, Paris 75013, France;; ^f^General Pediatrics, Infectious Disease and Internal Medicine Department, Robert Debré Hospital, Assistance Publique–Hôpitaux de Paris, Paris 75019, France;; ^g^Pediatric Pulmonary Department, Necker Hospital for Sick Children, Assistance Publique–Hôpitaux de Paris, Paris 75015, France;; ^h^Institut Necker-Enfants Malades, INSERM U1151, Paris 75015, France;; ^i^Institute for Human Genetics, University of California, San Francisco, CA 94143;; ^j^Pediatric Radiology, Necker Hospital for Sick Children, Assistance Publique–Hôpitaux de Paris, Paris 75015, France;; ^k^Department of Virology, Necker Hospital for Sick Children, Assistance Publique–Hôpitaux de Paris, Paris 75015, France;; ^l^Department of Virology, INSERM, Infection, Antimicrobiens, Modélisation, Evolution, UMR 1137, Bichat-Claude Bernard Hospital, University of Paris, Assistance Publique–Hôpitaux de Paris, Paris F-75018, France;; ^m^Department of Virology, Cochin Hospital, University of Paris, Assistance Publique–Hôpitaux de Paris, Paris 75014, France;; ^n^Institute for Human Genetics, University of California, San Francisco, CA 94143;; ^o^Departments of Epidemiology and Biostatistics and Bioengineering and Therapeutic Sciences, University of California, San Francisco, CA 94143;; ^p^Division of Rheumatology, Department of Medicine, University of California, San Francisco, CA 94143;; ^q^Parker Institute for Cancer Immunotherapy, San Francisco, CA 94129;; ^r^Chan Zuckerberg Biohub, San Francisco, CA 94158;; ^s^HHMI, The Rockefeller University, New York, NY 10065

**Keywords:** COVID-19, SARS-CoV-2, inherited primary immunodeficiency, interferon, RNA-seq

## Abstract

We describe an unvaccinated child at risk for life-threatening COVID-19 due to an inherited deficiency of IRF9, which governs ISGF-3–dependent responses to type I and III interferons (IFN). She was admitted, with a high nasal SARS-CoV-2 load on day 1 of upper respiratory tract infection. She was viremic on day 2 and received casirivimab and imdevimab. Her clinical manifestations and viremia disappeared on days 3 and 4, respectively. Circulating SARS-CoV-2 virus induced the expression of IFN-stimulated genes in leukocytes on day 1, whereas the secretion of blood type I IFNs, which peaked on day 4, did not. Antibody-mediated SARS-CoV-2 neutralization is, therefore, sufficient to overcome a deficiency of antiviral IFNs.

## Significance

Life-threatening COVID-19 pneumonia can be caused by rare inborn errors of type I interferon (IFN) immunity, or by autoantibodies neutralizing IFN-α2 or IFN-ω. In 2018, we reported a girl with critical influenza pneumonia due to inherited IRF9 deficiency, a component of the ISGF-3 transcription factor. We report the course of COVID-19 in the same patient. She was admitted on day 1 of upper respiratory tract infection with viremia. Administration of SARS-CoV-2–specific neutralizing monoclonal antibodies on day 2 prevented the development of pneumonia. SARS-CoV-2–specific monoclonal antibodies were sufficient to overcome a lack of ISGF-3– and IRF9-dependent type I and type III IFN immunity to the virus. They should be considered in selected children at high risk of life-threatening COVID-19.

Interindividual clinical variability in the course of SARS-CoV-2 infection is huge, ranging from silent infection to fatal COVID-19 (1). We have reported that life-threatening COVID-19 pneumonia can be caused in about 20% of cases by rare inborn errors of Toll-like receptor (TLR)3-, TLR7-, or IRF7-dependent type I interferon (IFN) immunity (2, 3), or by the presence of autoantibodies (auto-Abs) neutralizing IFN-α2 or IFN-ω, or more rarely IFN-β (4–16). These auto-Abs were long known to exist but were previously thought to be clinically silent. In contrast, recessive and dominant inborn errors of the type I IFN-inducing and response pathways had previously been reported in patients with other severe viral illnesses, including severe influenza pneumonia (17–20). Surprisingly, we found within the population of patients with critical COVID-19 pneumonia and inborn errors of type I IFN immunity, four unrelated and previously healthy adults, aged 25 to 50 y, with autosomal recessive (AR) IRF7 (*n* = 2) or IFNAR1 (*n* = 2) deficiency. The absence of IRF7 prevents the amplification of type I and III IFNs, whereas that of IFNAR1 prevents cellular responses to type I IFNs (and their ensuing amplification). These findings revealed that patients with inborn errors of type I IFN immunity, particularly those with recessive deficiencies of IRF7 or IFNAR1, whose biochemical defects are complete, are at high risk of life-threatening COVID-19 pneumonia (21). They further suggested that the early administration of IFN-α or -β in the course of SARS-CoV-2 infection might benefit patients with inborn errors impairing the production of type I IFNs (22), whereas IFN-β administration may be beneficial in patients with neutralizing auto-Abs against IFN-α but not IFN-β (23, 24). Moreover, these findings suggested that patients with inborn errors of the type I IFN response pathway or with auto-Abs neutralizing both IFN-α and IFN-β should be treated differently, perhaps with monoclonal antibodies (mAbs) against SARS-CoV-2 (25–27).

In 2018, we reported AR complete IRF9 deficiency in a 5-y-old French girl of Algerian ancestry (28). At the age of 2 y, she was admitted to an intensive care unit for severe influenza pneumonia due to influenza A virus (IAV). She required mechanical ventilation for 6 d for acute respiratory distress syndrome (ARDS) (28). She has since received prophylactic intravenous IgG every 3 wk and has been vaccinated annually against influenza, which has considerably improved her clinical status, as she has developed no other severe viral illness. IRF9 is a crucial component of the type I and III IFN signaling pathways, as it associates with STAT1 and STAT2 to form the trimeric ISGF-3 transcription factor, which governs cellular antiviral responses to type I and III IFNs (29–31). We showed that the c.991G > A mutant *IRF9* allele in the patient was loss-of-function, resulting in a lack of both ISGF-3 activation and ISGF-3–dependent IFN stimulated gene (ISG) induction following the stimulation of the patient’s cells with IFN-α2 (28). Accordingly, the patient’s fibroblasts displayed a high degree of susceptibility to IAV infection, which was not rescued by pretreatment with IFN-α2. Antiviral immunity was broadly impaired in vitro, as the patient’s cells also displayed impaired cell-intrinsic immunity to parainfluenza virus and respiratory syncytial virus. In the context of our discovery of inborn errors of type I IFN underlying critical COVID-19 pneumonia (2–4, 21), these findings strongly suggested that this patient was at high risk of life-threatening COVID-19 pneumonia. The lack of ISGF-3 activation in response to both type I and type III IFNs, an immunological phenotype even more severe than that of patients with AR IRF7 deficiency, whose cells produce IFN-β but do not amplify either type I or type III IFNs (19), further suggested that this patient might even develop a fulminant form of COVID-19 (19, 28). However, the parents refused to have their 8-y-old daughter vaccinated against SARS-CoV-2.

**Fig. 1.**
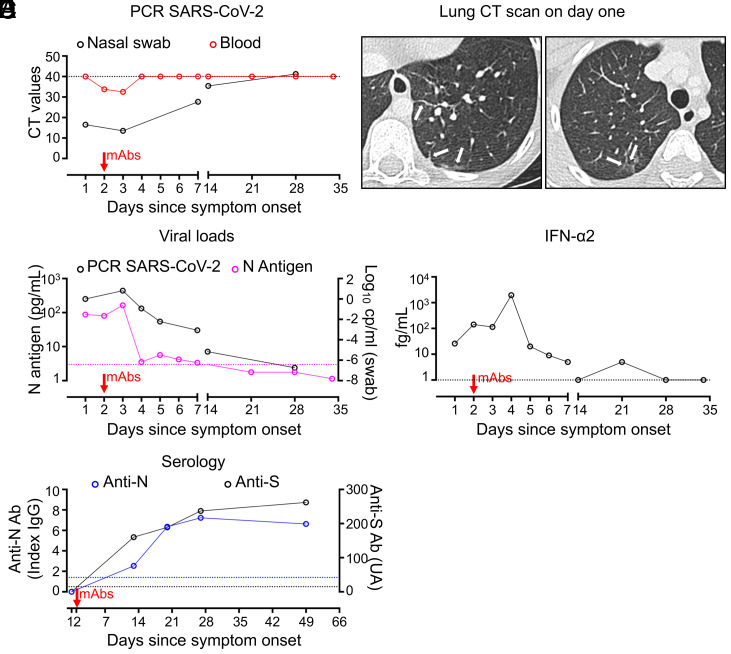
(*A*) RT-PCR for SARS-CoV-2 on nasal swabs and blood on the day of symptom onset (day 1), and at various time points following treatment with casirivimab and imdevimab (mAbs), prescribed on day 2 of symptoms. (*B*) CT of the lungs of the patient on day 1, showing bilateral curvilinear subpleural opacities. (*C*) Decrease in viral load, in log_10_ copies per milliliter, in nasal swabs and decrease in N-antigenemia (in pg/mL) following treatment with mAbs. (*D*) IFN-α2 determination by Simoa, on plasma collected from the patient on day 1, and at various time points after mAb treatment. (*E*) Serological results for SARS-CoV-2 anti-nucleocapsid protein (N) and spike protein (S) antibodies in the patient’s serum at various time points after the initiation of mAb treatment.

She was admitted to our unit on the first day of clinical manifestations consistent with mild, upper respiratory tract COVID-19, including rhinitis, cough, fatigue, and low-grade fever (38.2 °C). On day 1, PCR on a nasal swab revealed a very high load of SARS-CoV-2 (Ct: 16.5; 8.4 log_10_ copies per milliliter) and showed the patient to be infected with the N501Y α-variant (previously known as B.1.1.7) (32). SARS-CoV-2 was undetectable in the blood on day 1, but viremia was detected on day 2 (Fig. 1*A*). Oxygen saturation was 97%, respiratory rate was 20 breaths per minute, and physical examination found diminished breath sounds in the upper right lobe, consistent with previous examinations. Complete blood count was normal and blood C-reactive protein concentration was 7 mg/L. A computed tomography (CT)-scan of the lungs on day 1 revealed bilateral curvilinear subpleural opacities, and excluded a moderate (i.e., nonhypoxemic) form of COVID-19 pneumonia, as all lesions were already present on a CT-scan at 5 years of age, consistent with sequelae of the patient’s prior influenza ARDS (Fig. 1*B*).

On day 2, the patient received a single half-dose (600 mg) of intravenous casirivimab and imdevimab, a combination of human IgG1 neutralizing the receptor-binding domain of the SARS-CoV-2 spike protein (25, 33). Although there are currently no recommendations for administration of casirivimab and imdevimab in children (26), the strong rationale for this treatment was: 1) its recommended use in patients at high risk of critical COVID-19 pneumonia but not yet requiring oxygenotherapy (25, 34); 2) its efficacy in patients with high nasopharyngeal viral loads, as in this patient (25); 3) the known vulnerability of this IRF9-deficient patient and her cells to various respiratory viruses (28); 4) the pulmonary sequelae of influenza ARDS in this patient (28); 5) the occurrence in this patient of SARS-CoV-2 viremia, which is associated with a high risk of respiratory deterioration and death (35); 6) the high risk of hypoxemic COVID-19 pneumonia in patients with AR IFNAR1 or IRF7 deficiency (2, 19); and 7) the lack of a better therapeutic option, as therapy with IFN-α or -β was predicted to fail in the absence of ISGF-3 (as would treatment with IFN-λ, had it been available).

The patient did not receive any other medication, her symptoms and signs disappeared on day 3, and she was discharged on day 8, after a second CT-scan found no new pulmonary lesions. The most recent follow-up evaluation on day 50 was unremarkable; the patient was completely asymptomatic. Regular monitoring, by viral PCR on nasal swabs and blood, showed that SARS-CoV-2 viremia resolved on day 4 (Fig. 1*A*), whereas nasal viral load decreased strongly on day 7 (−3.06 log_10_ copies per milliliter; Ct: 27.7; 5.3 log_10_ copies per milliliter) (Fig. 1 *A* and *C*), reaching the threshold of detection on day 14 (Ct: 35.4; 3.2 log_10_ copies per milliliter). SARS-CoV-2 antigenemia peaked on day 3, with antigens undetectable from day 4 onward (Fig. 1*C*). The production of IFN-α2, as assessed by Simoa on plasma from the patient, was detectable on day 1 (26 fg/mL) and within the range reported in patients with COVID-19 not requiring mechanical ventilatory support (10 to 70,930 fg/mL; median 3,240 fg/mL) (36). It increased until day 4 (1,951 fg/mL), and then sharply decreased on day 5 (20 fg/mL), becoming undetectable by day 14 (Fig. 1*D*). We determined the levels of anti–SARS-CoV-2 antinucleocapsid protein (N) antibodies in the serum of the patient, to assess her own antibody response to SARS-CoV-2, in the presence of circulating casirivimab and imdevimab. The patient had detectable anti-N antibodies in her serum on day 14, and their titers were found to have increased on days 21 and 28 (Fig. 1*E*).

**Fig. 2.**
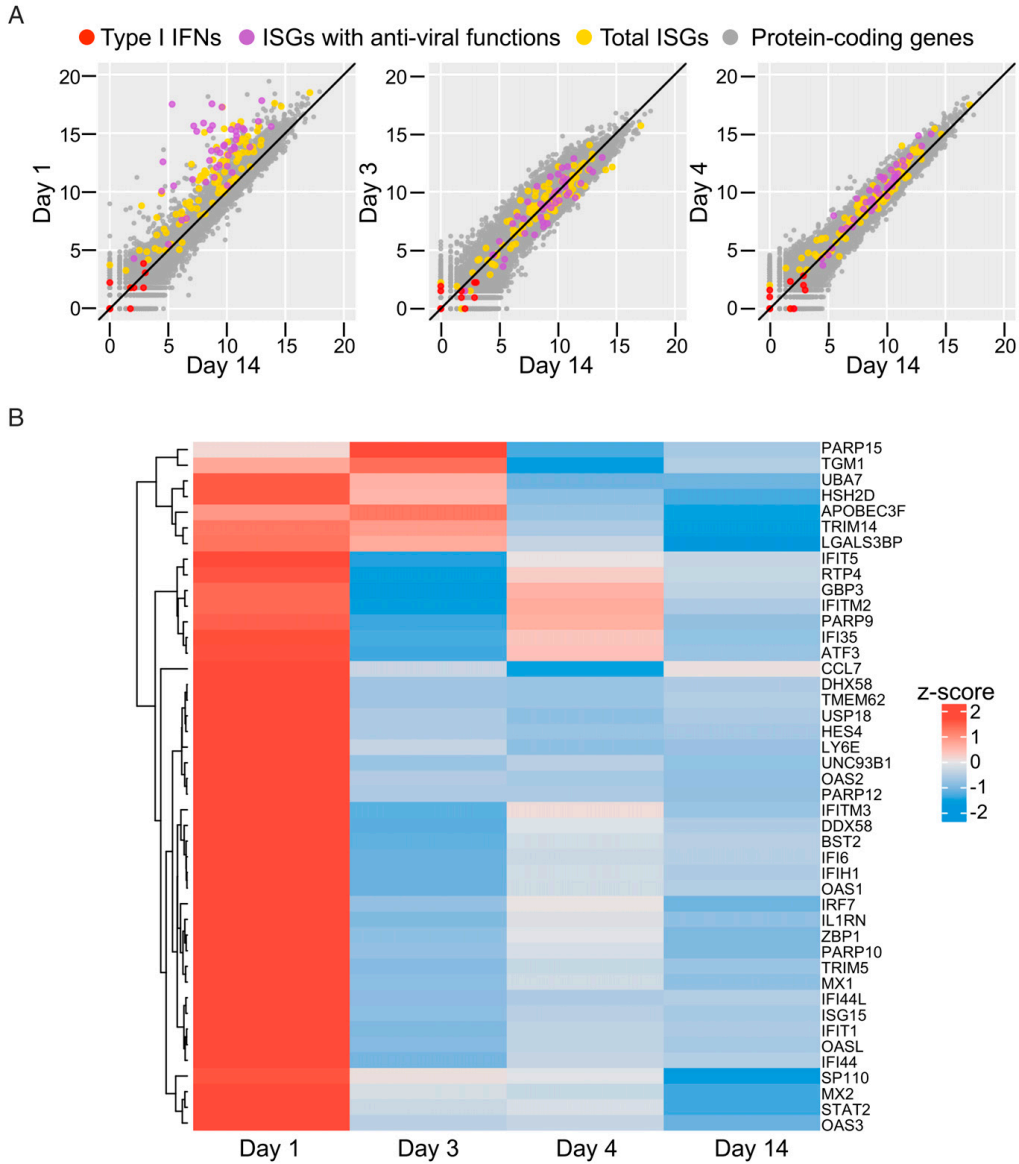
(*A*) RNA-seq comparison of gene expression between day 1, day 3, day 4, and day 14 in the patient, with expression assessed as the normalized and log-transformed read count (red: type-I IFN genes; purple: ISGs known to have antiviral functions; yellow: other ISGs; gray: other protein-coding genes). (*B*) Heatmap of RNA-seq gene expression *z*-scores for 44 ISGs with known antiviral functions in the patient on day 1, day 3, day 4, and day 14.

We also collected longitudinal whole-blood samples from the patient at various time points from day 1 to day 14. We performed whole-blood RNA sequencing (RNA-seq) with hemoglobin RNA depletion. We analyzed the transcripts of genes involved in antiviral immunity. We found that type I IFN transcripts, including that for IFN-β, were undetectable or only detectable in very low amounts at all time points, including days 1 to 4, although IFN-α2 protein was detected in the serum by Simoa (Fig. 2*A*). The source of the IFN-α2 in the patient’s blood may have been the upper respiratory tract (37). Furthermore, the frequency of plasmacytoid dendritic cells (pDCs) among leukocytes may be too low for the detection of their intrinsic type I IFN mRNA production by whole-blood RNA-seq. Either way, the absence of detectable type I IFN transcripts in the patient’s leukocytes is consistent with a lack of autoamplification of type I IFNs in the absence of IRF9 and ISGF-3. However, we found that the levels of transcripts of 140 previously defined type I IFN ISGs (5), 44 of which are known to be antiviral (38), were high before mAb therapy (Fig. 2). This is consistent with the initial sensing of viral infection by pDCs (39). These ISGs are probably triggered by the virus via IRF3, IRF5, and IRF7, but not IRF9 (which binds ISRE DNA motifs in ISGF-3 complexes). Moreover, no ISG induction was observed on days 3 and 4, despite a strong increase in serum IFN-α2 protein levels, consistent with the abolition of type I IFN signaling in the blood cells of the patient (Fig. 2). Thus, our findings indicate that SARS-CoV-2 induced ISRE-driven ISGs in leukocytes via transcription factors other than the IRF9-containing ISGF-3—presumably therefore via IRF3, IRF5, and IRF7—whereas leukocytes displayed no type I IFN induction (other than possibly in pDCs) and did not respond to type I IFNs made by other cells or tissues, such as pDCs and upper respiratory tract tissues. We further compared our patient’s ISGs signature (*z*-score) to that of other patients infected with SARS-CoV-2, hospitalized within the first week since symptom onset, and sampled longitudinally (5) (*SI Appendix*, Fig. S1). Patients whose medical state deteriorated and who succumbed all had a high ISG *z*-score early during COVID-19 or before death, whereas patients who survived had a lower ISG *z*-score along the course of recovery. Our patient’s very high ISG *z*-score on day 1 therefore further suggested that she might have developed a life-threatening form of COVID-19 had she not received the mAbs neutralizing SARS-CoV-2.

We, thus, report the safety and efficacy of combined casirivimab and imdevimab therapy for preventing the development of pneumonia and life-threatening COVID-19 in a child with AR complete IRF9 deficiency whose cells do not activate ISGF-3 or display induction of the ISGs activated by this transcription factor in response to both type I and III IFNs. The efficacy of antibody-mediated viral neutralization in the patient was demonstrated by the rapid resolution of her clinical manifestations. Further evidence of efficacy was provided by: 1) the rapid decrease in nasal viral load, as reported in other patients (25); and 2) the rapid clearance of SARS-CoV-2 viremia, which was initially predictive of a severe outcome (35). Patients with AR IRF9, STAT1, or STAT2 deficiencies, and disrupted cellular responses to both type I and III IFNs, and patients with IFNAR1 or IFNAR2 deficiencies may benefit from the same approach. It is unclear whether patients with AR interleukin-10RB deficiency, resulting in a lack of cell response to type III but not to type I IFNs, are at risk for severe COVID-19. Moreover, patients with auto-Abs neutralizing not only IFN-α and -ω, but also IFN-β, might also benefit from this approach, whereas IFN-β (alone or in combination with mAbs) may remain a treatment of choice in the absence of detectable IFN-β–neutralizing auto-Abs (23, 40). This observation further suggests that children with inborn errors of, or auto-Abs to type I IFNs, should be vaccinated against SARS-CoV-2. This observation is also important for the general population, as it provides proof-of-concept that mAbs specific for SARS-CoV-2 can efficiently prevent critical pneumonia, even in the patients most vulnerable to SARS-CoV-2, with inheritably compromised type I and III IFN immunity.

## Material and Methods

### PCR on Nasal Swabs and Blood.

PCR was performed on nasal swabs with the Alinity kit (Abbott). PCR was performed on blood with the SIMPLEXA COVID-19 direct assay (DiaSorin).

### Antigenemia.

Serum N antigen concentrations were determined with the COV-QUANTO ELISA kit (AAZ).

### IFN-α2 Determination by Simoa.

Serum IFN-α2 concentrations were determined with Quanterix platforms, as previously described (36).

### RNA-seq.

We collected whole blood from the patient on days 1, 3, 4, and 14, with a PAXgene Blood RNA tube. The blood sample was subjected to hemoglobin RNA depletion. Samples were sequenced on the Illumina NextSeq platform with a single-end 75-bp configuration. The RNA-seq fastq raw data were inspected to ensure that they were of high quality. The sequencing reads were mapped onto the human reference genome GRCh38 with STAR aligner v2.7, and the mapped reads were then quantified to determine the gene-level read counts, with featureCounts v2.0.2. The gene-level read counts were normalized and log_2_-transformed with DESeq2, to obtain the gene expression profile for all samples.

### Patient Recruitment and Ethics.

Clinical history and biological specimens were obtained from the referring clinicians, upon verification that a signed consent was available for each participant in the study. All the experiments involving human subjects conducted in this study were performed in accordance with institutional, local, and national ethical guidelines. Approval was obtained from the French Ethics Committee “Comité de Protection des Personnes,” the French National Agency for Medicine and Health Product Safety, the “Institut National de la Santé et de la Recherche Médicale,” in France (protocol no. C10-13, ID-RCB no. 2010-A00634-35), and the Rockefeller University Institutional Review Board in New York (protocol no. JCA-0700).

## Data Availability

All study data are included in the article and *SI Appendix*. The whole blood RNA-sequencing data are available in NCBI-SRA database, under project ID PRJNA770903.
